# A Favourable Termination of an Adhesion of the Placenta after Delivery

**Published:** 1800-01

**Authors:** H. Davies

**Affiliations:** Piccadilly


### *To the Editors of the Medical and Phyſical Journal.*

GENTLEMEN,

IF you think the incloſed caſe worthy a place in the Medical and Phyſical Journal, by its inſertion you will oblige,

GENTLEMEN,

Your's, reſpectfully,

*Piccadilly, Sept.* 12, 1799. H. DAVIES.

---

### *A favourable Termination of an Adheſion of the Placenta after Delivery.*

A lady, aged about forty years, of a leucophlegmatic habit, was taken in labour on Friday, June 9, 1799, of her fifth or ſixth child. Upon inquiry into the nature of the preſentation, it was found to be natural, though at this time the child was very high up in the pelvis. The pains continued (though languid) during that night, on Saturday, and the following day till the evening, when they altered from what might be termed *preparatory*, to the *expulſatory* ones, and the patient was delivered, about eight o'clock, of an uncommonly fine and healthy child. After dividing the funis, according to my uſual cuſtom, I waited about a quarter of an hour (the patient being conſiderably exhauſted) before I proceeded to deliver the placenta. The funis, in this caſe, was unuſually large, and diſtended with blood. Upon a moderate degree of preſſure, with a view to the extraction of the placenta, I ſoon diſcovered that it was diſpoſed to give way, while, at the ſame time, the placenta itſelf remained immoveable; and on continuing my efforts, though in the moſt gentle manner, it ſeparated at its inſertion into the placenta. Having loſt this aid to the extraction, and an hæmorrhage of too conſiderable a nature taking place to truſt it to the natural efforts of the ſyſtem, I endeavoured to lay hold of the ſubſtance of it, and bring it away. Herein I was alſo foiled; for I found the adheſion ſo conſiderable, the texture texture of the placenta so infirm, and the exhaustion of my patient so great, as not to justify persisting any longer in my efforts to remove it. It occurred to my mind here, that probably, by giving the patient a little respite, supporting her with gentle cordials, &c. I might promise myself better success at a little distance of time. But finding the hæmorrhage rather alarming, and the patient sinking, I resolved, in less *than an hour* from the first attempt, to make another effort, as the only alternative left to preserve her life; being well aware that the hæmorrhage would not cease, whilst the substance of the placenta was distending the cavity of the uterus. Upon the introduction of the hand, I found the placenta in the upper part of the uterus, and bending my fingers, I thrust them into its substance, determining, if practicable, to bring it away entire; or, as much of it as I possibly could. Here again my most sanguine expectations were cruelly disappointed, for I found, notwithstanding I had so firm a hold of it, it gave way, and only a portion of it could be extracted; the remainder adhering to the uterus. From the now unusually exhausted state of my patient, I thought it unwarrantable to proceed in my attempts any further. Repeated faintings, colliquative sweats, and a small pulse, not to be counted, were symptoms, in my opinion, of too alarming a nature to permit the continuance of an operation which was calculated to aggravate them; I therefore trusted the consequence to medical treatment only, which I hoped would assist the powers of the system to accomplish that naturally which could not be done artificially; and herein I am happy to say, my plan was crowned with success; for in a few days (three at farthest) the expulsion of the remaining portion, naturally took place by the efforts of the constitution, and the patient happily recovered. As it may not be unprofitable to mention the plan that was adopted upon this occasion, it consisted of the following mode of treatment: During the great exhaustion after the operation, I ordered a volatile draught to be given every two hours, and in the intervals to administer cordials, such as weak brandy and water, wine, &c. which, in the course of a few hours, had the happiest effects, insomuch that the sinking pulse was considerably raised, the pallid countenance materially altered, and the whole system partook of the general benefit. But, still I was aware that the constitution must be further invigorated, in order to accomplish the wished-for event, viz. of expelling the remainder of the placenta; I therefore ordered the abdomen to be fomented frequently, a warm clyster to be administered occasionally, and a strong preparation of the cortex and bitters as often as the stomach would bear; and, in *three days* from the first attempt, I had the pleasure

ſure to find that the expulſion took place. I now conſidered my patient in a ſtate of convaleſcence; continued my tonic plan, with the addition of chalybeate preparations to the bitters; and in a *fortnight* from the delivery, I had the happineſs of taking my leave of her, her health being ſo far reſtored as to ſupercede the neceſſity of any further medical treatment.

I ſhould not have troubled the medical world with the narration of the above caſe, had I not read in a late publication of a ſimilar one, where it appeared to me, that manual efforts in extracting the placenta had been too forcibly and prematurely perſiſted in; and the cordial, tonic plan, too long protracted. If the treatment above deſcribed ſhould prove uſeful in a correſponding caſe, I hope it will form a ſufficient apology for its inſertion in the Medical and Phyſical Journal, and the writer will be amply gratified.

